# Chew and spit (CHSP) in bariatric patients: a case series

**DOI:** 10.1186/s40337-021-00441-5

**Published:** 2021-07-21

**Authors:** Phillip Aouad, Kristin Stedal, Gro Walø-Syversen, Phillipa Hay, Camilla Lindvall Dahlgren

**Affiliations:** 1 InsideOut Institute, University of Sydney, Sydney, Australia; 2 School of Medicine, Western Sydney University, Penrith, NSW Australia; 3 Regional Department for Eating Disorders, Oslo University Hospital, Oslo, Norway; 4 Translational Health Research Institute, Western Sydney University, Penrith, NSW Australia; 5 Department of Psychology, Bjørknes University College, Oslo, Norway

**Keywords:** Case report, Case series, Chew and spit, CHSP, Bariatric, Weight loss surgery

## Abstract

**Background:**

Studies into the disordered eating behaviour of chew and spit have alluded to several cohorts more likely to engage in the behaviour, one such group being bariatric surgery candidates and patients. Weight-loss surgery candidates have received little to no attention regarding engaging in chew and spit behaviour. Changes in pre- and post- surgery eating pathology related to chew and spit behaviour has yet to be explored and described in academic literature.

**Case presentation:**

The current study reports on three cases of individual women, aged 30, 35, and 62 respectively, who indicated engagement in chew and spit. All three cases underwent bariatric surgery (two underwent gastric bypass, one underwent vertical sleeve gastrectomy). Eating pathology—including chew and spit behaviour, anxiety and depression, and adherence to the Norwegian nutritional guidelines were examined pre-operatively and post-operatively (one and two-year follow-up). At baseline (pre-surgery), two participants reported that they engaged in chew and spit, compared to one patient post-surgery. All three cases reported that they, to at least some extent, adhered to dietary guidelines post-surgery. Subjective bingeing frequency appeared to be relatively low for all three cases, further declining in frequency at one-year follow-up. At baseline, one participant reported clinically significant depression and anxiety, with no clinically significant depression or anxiety reported at follow-ups in participants that chew and spit.

**Conclusions:**

The current study provides a starting point for the exploration of chew and spit as a pathological symptom of disordered eating in bariatric patients. It highlights the need to further explore chew and spit before and after weight-loss surgery.

## Introduction

Weight-reduction procedures have been increasing in popularity in recent years [8]. Bariatric surgery is a procedure performed to aid patients in achieving weight loss and to minimize risk of obesity-related health complications [25]. Weight loss is achieved by one of two mechanisms: the first mechanism being a reduction in stomach capacity by removing a substantial portion of the stomach, such as Vertical Sleeve Gastrectomy (VSG). The second mechanism is through malabsorption, where part of the intestine is bypassed so that less absorption occurs, such as Roux-en-Y gastric bypass [25].

Most often, bariatric surgery is recommended for individuals who have a BMI over 40 kg/m^2^ (or > 35kg/m^2^ with medical comorbidity) who have otherwise been unable to lose weight or maintain weight loss using conventional methods such as behavioural and pharmacological treatments [25].

According to Williams-Kerver et al. Williams-Kerver, Steffen, & Mitchell [35] several disturbed eating behaviours and eating disorders (EDs) have been noted in individuals prior to bariatric surgery, which often significantly reduce post-operatively [5, 10, 22, 27, 30]. Of specific note, Bulimia Nervosa (BN) and associated compensatory behaviours (e.g. vomiting, misuse of laxative and diuretics), loss of control (LoC) eating (i.e. feeling unable to stop eating, or to control what and how much one eats), objective and subjective binge eating, and night eating are more prevalent in pre-operative versus post-operative bariatric patients [35]. Perhaps most prevalent among the pre-operative disorders in bariatric patients is Binge Eating Disorder (BED) [35].

Although disordered eating behaviours associated with loss of control (LoC) are particularly prevalent in bariatric surgery candidates, objective binge eating tends to drastically reduce post-operatively as patients will have reduced stomach capacity [24]. However, other pathological eating behaviours may go unnoticed due to the change in presentation of disordered eating pathology after surgery [32]. Recent studies have suggested certain disordered eating behaviours may increase postoperatively, leading to potential physical and psychological complications, and reduced adherence to post-operative eating guidelines [1, 35]. One such behaviour is “Chew and Spit” (CHSP). CHSP is a behaviour characterised by masticating food and expelling the contents of the mouth before swallowing [1]. CHSP is most often used as a weight control measure and allows the individual to taste the food without ingesting it fully.

Studies into CHSP [1] have reported individuals with EDs [2, 14, 23, 33], adolescents and young people [3], and those who may have particular dietary requirements due to surgical interventions or other medical necessity [1] are more likely to engage in CHSP behaviour. It is this last group that appears to have received least attention to date, in particular individuals who have had weight loss surgery.

A study conducted by D’Souza et al. [8] has investigated a range of pre- and postoperative disordered eating behaviours, which reduced within the two years prior surgery. However, no prior study has focused on the occurrence of CHSP, or its psychopathological correlates in bariatric surgery patients. Given its modest prevalence rate among the general population [2], the study of CHSP in bariatric patients who are prone to engage in disordered eating behaviours and have to adhere to strict postoperative dietary guidelines, is warranted. Therefore, the aim of this descriptive and exploratory study is to gain preliminary insight into CHSP in a bariatric sample. As the nature of the current study is exploratory, no specific hypotheses have been developed.

The identification of pathological eating behaviours in bariatric surgery treatment is important in order to identify subgroups of patients presenting with vulnerabilities that limit optimal weight loss if not addressed and treated properly in follow-up regimens. Relevant to the present study, these behaviours may arise and subsequently go unresearched due to limitations of utilised measurements [18, 19] or non-disclosure of eating pathology [20]. Importantly, given that no specific psychological intervention has been developed to target CHSP, nor have existing treatments been tested in individuals who CHSP let alone in bariatric surgery patients, it is paramount that we understand if, and how, CHSP is implicated in worsening eating pathologies pre- and post- operatively.

Given the limited knowledge base surrounding CHSP and bariatric surgery, as well as the potential hampering of weight-loss efforts in individuals who have undergone bariatric surgery [35] the primary aim of the current study was to explore and describe the phenomena as it may appear in bariatric patients before and after surgery, and to provide directions for future research. The current report examines three individuals who have undergone bariatric surgery and who have admitted to engaging in CHSP behaviour either before or after their procedure. This case series follows the CARE Guidelines for case reporting [31].

## Assessment

The following measures were used to assess patients pre- and post- operatively [34]:

### EDE-Q

Prior to undergoing bariatric surgery, participants’ eating pathology and attitudes were assessed using three items (13, 14, & 15) of the Eating Disorder Examination Questionnaire (EDE-Q; [7]. The EDE-Q measures disordered eating behavior in the preceding 28-days, and is a self-report measure of severity and frequency of behaviors using a 7-point Likert scale (0 = No days/Not at all to 6 = Everyday/Markedly), with higher scores reflecting stronger severity and duration over the previous 28-days [26]. The three items used from the EDE-Q included looking at frequency of overeating eating (item 13), frequency of subjective Loss of Control during an overeating episode (LoC,item 14), and the number of days (items 13 and 14 indicated ‘number of episodes’, not number of days—meaning participants could report multiple episodes a day) participants experienced both overeating and LoC (item 15, [12]. In the current study, the three-items used for this measure had excellent internal reliability for both males (α = 0.87) and females (α = 0.87) (total α = 0.82) [7], [11].

### EDE-Q: BSV (CHSP frequency)

Once participants had undergone surgery (12 months post-operative), eating pathology was assessed using the Eating Disorder Questionnaire: Bariatric Surgery Version (EDE-Q: BSV; [9, 13, 29]. Similar to the standard EDE-Q, the EDE-Q: BSV measures ED behavior across five subscales – dietary restraint, eating concerns, shape/weight overvaluation, and appearance concern—over the past 28-days, with participants rating the severity and frequency of pathological eating behaviors on a 7-point Likert scale [9]. Although the EDE-Q: BSV is closely related to the standard EDE-Q, it more accurately and comprehensively assesses eating pathology in a bariatric population by focusing on post-operative behaviors. Notably the EDE-Q: BVS compares the presence of pre- and post- operative BED and Bulimia Nervosa [9], [17].

The predominant purpose of the EDE-Q: BSV was to assess CHSP frequency and severity in the 28-days prior to participating in the study. Using an adapted version (Appendix A) of the eight items contained in the EDE-Q: BSV, participants answered three CHSP questions prior to surgery including:

1. Over the past four weeks have you chewed food and spit it out without swallowing it to influence your weight or shape (‘yes’ or ‘no’ response);
2. If yes, how many times have you done this in the past 28-days?;
3. How distressed have you been about chewing and spitting (0 = not at all; 6 = extreme).

Participants were asked all eight items related to CHSP at one-year follow up. It should be noted that due to participants having to follow a strict pre-surgery diet, which involves liquid protein drinks and little to no solid foods 3-4 weeks prior to surgery, question 1 and 2 of the EDE-Q: BSV (CHSP section) were modified from the original version. Question 1 asked participants to indicate the presence or absence of CHSP, while question 2 did *not* request participants to list the foods consumed instead asked for an indication of CHSP frequency.

### Hospital Anxiety and Depression Scale (HADS)

The Hospital Anxiety and Depression Scale (HADS) is a 14-item measure of both anxiety (7-items) and depression (7-items) symptomology, using a four-point Likert scale (from 3 = Yes, definitely to 0 = No, not at all); with questions 7 and 10 being reverse scored [36]. Higher scores typically signify increased anxiety and depression symptomology,with domain scores ≥ 11 indicating clinical levels of anxiety or depression, and ≤ 7 indicating no anxiety or depression [21]. The HADS has been well validated and in the current study, the measure had excellent internal reliability for both anxiety (α=0.85) and depression (α = 0.76) and had a total scale α of 0.88 at baseline.

### Adherence to guidelines

Patient observation of the *Norwegian National Dietary Guidelines* [28] were assessed by indicating on a Likert scale what level (from 1 ‘some,’ to 7 ‘a lot’) of perceived adherence the patient had for different guideline items, including: 1. not going 3-4 hours between each meal,2. choosing low fat meat products; 3. choosing low fat dairy products; 4. using wholegrain products; 5. eating five portions of fruit/vegetables per day; and 6. limiting their intake of fat and sugar. The six guidelines asked of participants, were a condensed version of the full 12 items, which also included items on physical activity [15], [16].

## Narrative

### Case presentation: patient-1

Patient-1 was a female aged 30 years old. Prior to vertical sleeve gastrectomy (VSG) surgery, her BMI was 41.5 kg/m^2^. Additionally, no prior lifetime or current psychiatric conditions were reported. Reported medicated comorbidities for patient-1 included hypothyroidism. It should be noted that patient-1 dropped out of the study at year-two follow-up.

Pre-operatively, patient-1 indicated engaging in CHSP twice in the 28 days prior to surgery with moderate distress attributed to the behaviour. CHSP had ceased at one-year follow-up. Binge eating frequency reduced significantly at one-year follow up (from score 6 to 1). The frequency and days of feeling a LoC increased at follow-up but were still considered 'low' (3 out of 28 days); Patient-1 indicated a strong adherence to national dietary guidelines pre-operatively, which increased marginally at follow-up. Anxiety and Depression symptomatology was well below clinical threshold both before and after surgery.

Scores are presented in Table 1.

**Table 1:** Patient reported scores related to binge eating, CHSP, adherence to national dietary guidelines, and anxiety and depression symptomatology

	Case	Baseline	1year	2year	
Guideline 1: meals 3-4hr apart	1	6	N/A	N/A	Adherence to dietary guidelines
	2	1	5	3	
	3	2	5	6	
Guideline 2: meat w less fat	1	6	7	N/A	
	2	7	6	3	
	3	7	7	5	
Guideline 3: dairy w less fat	1	N/A	5	N/A	
	2	5	5	2	
	3	5	7	6	
Guideline 4: grain products	1	6	5	N/A	
	2	7	6	5	
	3	5	7	5	
Guideline 5: five fruit vege day	1	6	5	N/A	
	2	1	2	1	
	3	1	1	1	
Guideline 6: limit fat sugar	1	5	5	N/A	
	2	7	5	2	
	3	3	6	4	
Binge: frequency	1	6	1	N/A	Binge eating
	2	4	0	1	
	3	0	0	0	
Binge: LoC frequency	1	1	2	N/A	
	2	0	10	1	
	3	0	0	0	
Binge: LoC days	1	0	3	N/A	
	2	0	6	1	
	3	0	0	0	
Spit Q1: spit presence	1	Yes	No	N/A	CHSP
	2	No	Yes	Yes	
	3	Yes	No	No	
Spit Q2: spit frequency	1	2	0	N/A	
	2	0	10	4	
	3	N/A	0	N/A	
Spit Q3: spit distress	1	3	0	N/A	
	2	0	3	0	
	3	0	0	N/A	
Spit Q4: reason 1: avoid dump	1	N/A	No	N/A	
	2	N/A	No	No	
	3	N/A	N/A	No	
Spit Q5: reason 2: enjoy sense	1	N/A	No	N/A	
	2	N/A	No	No	
	3	N/A	N/A	No	
Spit Q6: reason 3: other	1	N/A	No	N/A	
	2	N/A	Yes	Yes	
	3	N/A	N/A	No	
Spit Q7: frequency of reason	1	N/A	1	N/A	
	2	N/A	10	7	
	3	N/A	N/A	N/A	
Spit Q8: CHSP distress	1	N/A	1	N/A	
	2	N/A	2	0	
	3	N/A	N/A	N/A	
HADS: global total	1	1	1	N/A	Anxiety and depression
	2	11	5	N/A	
	3	6	8	3	

Spit Q1-3 were asked at baseline, Spit Q4-8 were added at follow-ups. The presence, frequency, and distress as a result of CHSP was assessed at baseline using three items (CHSP presence, CHSP frequency, CHSP distress) of the EDE-Q:BSV, compared to using all 8-items of the EDE-Q:BSV to assess CHSP at follow-up time-points. Case 1 was lost to year 2 follow-up and did not provide response for baseline dietary guideline 3, nor, a response for year 1 dietary guideline 1. Case 3 indicated to engaging in CHSP at baseline (Spit Q1) but did not specify an amount at baseline (Spit Q3)

### Case presentation: patient-2

Patient-2 was a female aged 35 years old. Prior to Roux-en-Y Gastric Bypass (GBP) surgery, her BMI was 42.7 kg/m^2^. As part of the bariatric surgery screening process, patient-2 did not raise significant psychological or pathological eating concerns that warranted her exclusion from the procedure. Additionally, no prior lifetime or current psychiatric conditions were reported. No reported medicated comorbidities for patient-2 were noted.

Pre-operatively, patient-2 did not indicate any engagement in CHSP, however at one-year follow-up indicated to engaging in at least 10 episodes of CHSP in the 28-days prior. At two-year follow-up CHSP was still present, but had reduced to 4 episodes in the 28-days prior. Prior to surgery patient-2 indicated she had been engaging in 'moderate' bingeing (score of 4) but with no associated loss of control. Subjective bingeing ceased at one-year follow-up and at two-year follow-up had occurred only once in the prior 28-days. Number of days (10 out of 28) and severity of feeling a subjective LoC increased significantly but was still considered 'low' (3 out of 28 days); Patient-2 indicated a low adherence to national dietary guidelines pre-operatively, which increased to near perfect adherence at follow-ups. Anxiety and Depressive symptomatology was at non-clinical levels at follow-ups.

Scores are presented in Table 1.

### Case presentation: patient-3

Patient-3 was a female aged 62 years old. Prior to Roux-en-Y GBP surgery, her BMI was 39.25kg/m2. As part of the bariatric surgery screening process, patient-3 did not raise significant psychological or pathological eating concerns that warranted her exclusion from the procedure. Patient-3 had previously been treated for an unspecified psychiatric disorder as an outpatient and was on antidepressant medication. Additionally, patient-3 was taking opioids for pain management, as well as medications for hypertension and hyperlipidaemia.

Pre-operatively, patient-3 indicated engagement in an unspecified amount of CHSP which ceased entirely post-operatively and was not reported at both follow-up timepoints. It should be noted that although patient 3 indicated to the presence of CHSP at baseline, they did not provide an amount for the frequency which they engaged in the behaviour. This was due to the questionnaires being self-report and not all questions being made mandatory or a requirement of treatment -clinical and research data was collected separately, through different processes.

Prior to surgery patient-3 indicated no bingeing or associated loss of control. This was also the case post-operatively at both follow-up timepoints. Patient-3 indicated a moderate adherence to national dietary guidelines pre-operatively, which appeared to increase further ' at one-year follow-up but had dropped slightly at the two-year mark. Anxiety and depressive symptomatology were at non-clinical (score < 7) levels pre- and post-operatively.

Scores are presented in Table 1.

## Discussion

The primary purpose of the current exploratory study was to examine CHSP and its relation to binge eating, dietary guideline adherence, as well as to anxiety and depression in a sample of bariatric surgery patients. At baseline, two patients reported engaging in CHSP which ceased following surgery, whereas one de-novo case of CHSP appeared postoperatively. Scores for anxiety and depression were stable or reduced from baseline to one year-follow up. Binge eating (with associated LoC) increased at one-year follow-up for all three CHSP cases. All three participants reported increased adherence to dietary guidelines. At two-year follow up, overall binge eating was reduced and reported adherence to dietary guidelines also dropped in all three patients. Furthermore, considering the point-prevalence of CHSP in the general population of adults is 0.4% [2] and taking into consideration the small clinical sample size of the current exploratory study, there may likely be some occurrence of CHSP behaviour among a larger bariatric population, which future studies may wish to investigate further.

In spite of assurances of anonymity and confidentiality, the participants may have hesitated to fully disclose pathological eating behaviours, including CHSP, in fear of the consequences for their forthcoming and scheduled surgery. Further, fear of judgment may have limited disclosure both prior to surgery and at follow-up appointments, as recent research has demonstrated that discussing CHSP is considered exceptionally taboo, even amongst more general samples [4], and therefore may be frequently underreported.

Putatively, it may be posited that some bariatric patients may use CHSP as a pre or post-operative coping strategy. Specifically, they may utilise CHSP as a means to adhere to pre-surgical liquid diet conditions and during post-surgical follow-up in response to the fear of causing complications, such as rupturing, in the gastrointestinal tract. Pathological eating behaviour in the post-operative period has the highest predictive value in determining an individual’s weight loss after bariatric surgery [6]. CHSP may therefore impede weight loss in this patient group, but this has not been explored. CHSP may occur in the presence of LoC [4] and has even been suggested as a marker for ED severity [14]. Additionally, CHSP may present a ‘cathartic outlet’ [4] in lieu of an individual’s post-operatively limited ability to binge. However, our findings provide preliminary insight into CHSP occurring both pre- and post-surgery, and therefore warrants additional studies using larger samples.

### Limitations

A number of limitations were noted for the current study, which included single site data collection, rendering the ability to draw inferences and generalize results to a wider bariatric sample, not possible. Additionally, questions were entirely self-reported, with majority of questions not being ‘mandatory’, which led to missing data, particularly at baseline. This was despite clinical and research questions being collected independently of each other, and participants being reassured that research questionnaires had no bearing on the outcome of treatment or care received.

Further, other indicators of eating pathology were low in the cases explored in the current study. Such difference, when compared to parallel estimates found in the bariatric surgery literature, may indicate the possible presence of selection bias and underreporting. Further, the addition of five CHSP questions at follow-up meant that deeper insight into CHSP (based on EDE: BSV) could not be compared pre- and post- operatively, only post-operatively at the one- and two-year follow-ups.

### Clinical significance and future research

Research investigating CHSP in bariatric surgery patients is limited. The current study provides a preliminary starting point for the exploration of CHSP as a pathological symptom of disordered eating in this sample. Moreover, given that participants may have been hesitant to be forthcoming about their CHSP behaviour, careful consideration of the approach to understanding CHSP in a bariatric population should also be given. Nonetheless, the use of a screening tools such as the EDE: BSV may provide useful, straightforward, and efficient examination of eating pathology in bariatric surgery patients, and overall has meaningful clinical utility.

## Conclusion

When examining dietary guideline adherence, binge eating, CHSP, anxiety and depression in three cases drawn from a convenience sample of bariatric candidates, it was found that a small number of individuals reported engaging in CHSP behaviour (both pre-operatively, or post-operatively). Specifically, it was noted that in the bariatric surgery cases examined, CHSP occurred to some level both pre- and post-operatively. Future studies should examine CHSP in a larger and more robust sample.

## Data Availability

The datasets generated and/or analysed during the current study are not publicly available due to the on-going nature of the study. Data requests are at the discretion of the data owner and/or participating institutions. Initial requests may be sought by contacting the study lead Camilla.Lindvall.Dahlgren@bhioslo.no.

## Abbreviations

BED: Binge Eating Disorder
BMI: Body Mass Index (kg/m^2^)
BN: Bulimia Nervosa
CARE: Case Report Guidelines
CHSP: Chew and Spit
ED: Eating Disorder
EDE-Q: Eating Disorder Examination - Questionnaire
EDE-Q: BSV: Eating Disorder Examination - Questionnaire: Bariatric Surgery Version
GBP: Gastric Bypass
HADS: Hospital Anxiety and Depression Scale
LoC: Loss of Control
VSG: Vertical Sleeve Gastrectomy
